# Supplementary material for: Prediction of diabetic kidney disease risk using machine learning models: A population-based cohort study of Asian adults
Source: eLife. 2023 Sep 14;12:e81878. doi: 10.7554/eLife.81878 (PMC10531395; doi:10.7554/eLife.81878)
Supplement: Supplementary file 1. — (Table 1a) Characteristics of the preprocessed datasets A–F. (Table 1b) List of variables used for DKD prediction. (Table 1c) Median AUC [IQR] performance of the ML models using datasets A–F. (Table 1d) Median SN%/SP% performance of the ML models using datasets A–F. (Table 1e) Source data linked to Figure 3. (Table 1f) Top ML-selected predictors for incident DKD in each of the three ethnic groups by EN and RFE. (Table 1g) Baseline characteristics of SEED diabetic participants by ethnicity (n = 1365). [file elife-81878-supp1.docx]

| **Supplementary Table 1a. Characteristics of the preprocessed datasets A - F** | | | | | | | |
| --- | --- | --- | --- | --- | --- | --- | --- |
|  |  | Dataset A | Dataset B | Dataset C | Dataset D | Dataset E | Dataset F |
| Feature category | Traditional risk factors | **√** | **√** | **√** | **√** | **√** | √ |
|  | Extended risk factors |  | **√** | **√** | **√** | **√** | √ |
|  | Imaging parameters |  |  | **√** |  |  | √ |
|  | Genetic profiles |  |  |  | **√** |  | √ |
|  | Serum metabolites |  |  |  |  | **√** | √ |
| The number of features, *p* | | 7 | 29 | 40 | 105 | 252 | 339 |
| Sample size, *n* | | 1334 | 1364 | 1305 | 1040 | 1327 | 976 |
| The number of events (%) | | 158 (11.84) | 162 (11.88) | 157 (12.03) | 124 (11.92) | 155 (11.68) | 116 (11.89) |
| Column-wise imputation, % | | 0 | 5.43 | 5.06 | 5.19 | 6.71 | 7.58 |
| Row-wise imputation, % | | 0 | 9.09 | 9.09 | 3.67 | 9.77 | 7.29 |

| **Supplementary Table 1b. List of variables used for DKD prediction** | | | |
| --- | --- | --- | --- |
| **7 Traditional risk factors** | | | |
| Age | | Blood glycosylated hemoglobin (HbA1c) | |
| Sex | | Duration of diabetes | |
| Ethnicity | | Body mass index (BMI), kg/m^2^ | |
| Systolic blood pressure (SBP) | |  | |
| **22 Extended risk factors** | | | |
| Marital status | | Pulse pressure, mm Hg | |
| Education level (primary, secondary, high school and above) | | Blood Glucose level, mmol/L | |
| Monthly household income (SDG <1000, 1000-2000, ≥2000) | | Serum total cholesterol, mmol/L | |
| Housing status (2-, 3, 4,5-bedrooms and above) | | Serum HDL Cholesterol, mmol/L | |
| Alcohol consumption (never drinker, ever drinker) | | Serum LDL Cholesterol, mmol/L | |
| Current smoker (yes, no) | | Estimated glomerular filtration rate (eGFR) | |
| Hypertension status (yes, no) | | History of cardiovascular disease (CVD) | |
| Anti-hypertensive medication use | | Intraocular pressure (left) | |
| Anti-cholesterol medication use | | Intraocular pressure (right) | |
| Anti-diabetic medication use | | Average axial length (left) | |
| Diabetic retinopathy (yes, no) | | Average axial length (right) | |
| **11 Retinal imaging parameters** | | | |
| Branching angle-artery (BAa) | | Fractal dimension C (FxC) | |
| Branching angle-vein (BAv) | | Fractal dimension C-artery (FxCa) | |
| Branching coefficient-artery (BCa) | | Fractal dimension (FxCv) | |
| Branching coefficient-vein (BCv) | | Tortuosity-artery (sTorta) | |
| Central retinal arteriolar equivalent (CRAE) | | Tortuosity-vein (sTortv) | |
| Central retinal venular equivalent (CRVE) | |  | |
| **223 Blood metabolites (non-lipoprotein subclasses)** | | | |
| Lipoprotein particle size | Mean diameter for HDL particles | Fatty acids (%) | Ratio of 18:2 linoleic acid to total fatty acids |
|  | Mean diameter for LDL particles |  | Ratio of 22:6 docosahexaenoic acid to total fatty acids |
|  | Mean diameter for VLDL particles | Fatty acids by saturation | Monounsaturated fatty acids |
| Cholesterol | Esterified cholesterol |  | Omega-3 fatty acids |
|  | Free cholesterol |  | Omega-6 fatty acids |
|  | Remnant cholesterol (non-HDL, non-LDL -cholesterol) |  | Polyunsaturated fatty acids |
|  | Serum total cholesterol |  | Saturated fatty acids |
|  | Total cholesterol in HDL | Fatty acids by saturation (%) | Ratio of monounsaturated fatty acids to total fatty acids |
|  | Total cholesterol in HDL2 |  | Ratio of omega-3 fatty acids to total fatty acids |
|  | Total cholesterol in HDL3 |  | Ratio of omega-6 fatty acids to total fatty acids |
|  | Total cholesterol in IDL |  | Ratio of polyunsaturated fatty acids to total fatty acids |
|  | Total cholesterol in LDL |  | Ratio of saturated fatty acids to total fatty acids |
|  | Total cholesterol in VLDL | Glycolysis related metabolites | Citrate |
| Glycerides and phospholipids | Phosphatidylcholine and other cholines |  | Lactate |
|  | Ratio of triglycerides to phosphoglycerides | Inflammation | Glycoprotein acetylation |
|  | Serum total triglycerides | Aromatic amino acids | Phenylalanine |
|  | Sphingomyelins |  | Tyrosine |
|  | Total cholines | Branched-chain amino acids | Isoleucine |
|  | Total phosphoglycerides |  | Leucine |
|  | Triglycerides in HDL |  | Valine |
|  | Triglycerides in IDL | Other amino Acids | Alanine |
|  | Triglycerides in LDL |  | Glutamine |
|  | Triglycerides in VLDL |  | Histidine |
| Apolipoproteins | Apolipoprotein A-I | Ketone bodies | 3-hydroxybutyrate |
|  | Apolipoprotein B |  | Acetate |
|  | Ratio of apolipoprotein B to apolipoprotein A-I |  | Acetoacetate |
| Total fatty acids and saturation measures | Estimated degree of unsaturation | Fluid Balance | Albumin |
|  | Total fatty acids |  |  |
| Fatty acids | 18:2, linoleic acid |  |  |
|  | 22:6, docosahexaenoic acid |  |  |
| **223 Blood metabolites (Lipoprotein subclasses)** | | | |
| Cholesterol esters | in chylomicrons and extremely large VLDL | Cholesterol esters to total lipids ratio | in chylomicrons and extremely large VLDL |
|  | in IDL |  | in chylomicrons and large VLDL |
|  | in large HDL |  | in chylomicrons and medium VLDL |
|  | in large LDL |  | in chylomicrons and small VLDL |
|  | in large VLDL |  | in chylomicrons and very large VLDL |
|  | in medium HDL |  | in chylomicrons and very small VLDL |
|  | in medium LDL |  | in IDL |
|  | in medium VLDL |  | in large HDL |
|  | in small HDL |  | in large LDL |
|  | in small LDL |  | in medium HDL |
|  | in small VLDL |  | in medium LDL |
|  | in very large HDL |  | in small HDL |
|  | in very large VLDL |  | in small LDL |
|  | in very small VLDL |  | in very large HDL |
| Free cholesterol | in chylomicrons and extremely large VLDL | Free cholesterol to total lipids ratio | in chylomicrons and extremely large VLDL |
|  | in IDL |  | in chylomicrons and large VLDL |
|  | in large HDL |  | in chylomicrons and medium VLDL |
|  | in large LDL |  | in chylomicrons and small VLDL |
|  | in large VLDL |  | in chylomicrons and very large VLDL |
|  | in medium HDL |  | in chylomicrons and very small VLDL |
|  | in medium LDL |  | in IDL |
|  | in medium VLDL |  | in large HDL |
|  | in small HDL |  | in large LDL |
|  | in small LDL |  | in medium HDL |
|  | in small VLDL |  | in medium LDL |
|  | in very large HDL |  | in small HDL |
|  | in very large VLDL |  | in small LDL |
|  | in very small VLDL |  | in very large HDL |
| Phospholipids | in chylomicrons and extremely large VLDL | Phospholipids to total lipids ratio | in chylomicrons and extremely large VLDL |
|  | in IDL |  | in chylomicrons and large VLDL |
|  | in large HDL |  | in chylomicrons and medium VLDL |
|  | in large LDL |  | in chylomicrons and small VLDL |
|  | in large VLDL |  | in chylomicrons and very large VLDL |
|  | in medium HDL |  | in chylomicrons and very small VLDL |
|  | in medium LDL |  | in IDL |
|  | in medium VLDL |  | in large HDL |
|  | in small HDL |  | in large LDL |
|  | in small LDL |  | in medium HDL |
|  | in small VLDL |  | in medium LDL |
|  | in very large HDL |  | in small HDL |
|  | in very large VLDL |  | in small LDL |
|  | in very small VLDL |  | in very large HDL |
| Total cholesterol | in chylomicrons and extremely large VLDL | Total cholesterol to total lipids ratio | in chylomicrons and extremely large VLDL |
|  | in large HDL |  | in chylomicrons and large VLDL |
|  | in large LDL |  | in chylomicrons and medium VLDL |
|  | in large VLDL |  | in chylomicrons and small VLDL |
|  | in medium HDL |  | in chylomicrons and very large VLDL |
|  | in medium LDL |  | in chylomicrons and very small VLDL |
|  | in medium VLDL |  | in IDL |
|  | in small HDL |  | in large HDL |
|  | in small LDL |  | in large LDL |
|  | in small VLDL |  | in medium HDL |
|  | in very large HDL |  | in medium LDL |
|  | in very large VLDL |  | in small HDL |
|  | in very small VLDL |  | in small LDL |
|  |  |  | in very large HDL |
| Triglycerides | in chylomicrons and extremely large VLDL | Triglycerides to total lipids ratio | in chylomicrons and extremely large VLDL |
|  | in large HDL |  | in chylomicrons and large VLDL |
|  | in large LDL |  | in chylomicrons and medium VLDL |
|  | in large VLDL |  | in chylomicrons and small VLDL |
|  | in medium HDL |  | in chylomicrons and very large VLDL |
|  | in medium LDL |  | in chylomicrons and very small VLDL |
|  | in medium VLDL |  | in IDL |
|  | in small HDL |  | in large HDL |
|  | in small LDL |  | in large LDL |
|  | in small VLDL |  | in medium HDL |
|  | in very large HDL |  | in medium LDL |
|  | in very large VLDL |  | in small HDL |
|  | in very small VLDL |  | in small LDL |
|  |  |  | in very large HDL |
| Concentration | chylomicrons and extremely large VLDL particles | Total lipids | in chylomicrons and extremely large VLDL |
|  | IDL particles |  | in IDL |
|  | large HDL particles |  | in large HDL |
|  | large LDL particles |  | in large LDL |
|  | large VLDL particles |  | in large VLDL |
|  | medium HDL particles |  | in medium HDL |
|  | medium LDL particles |  | in medium LDL |
|  | medium VLDL particles |  | in medium VLDL |
|  | small HDL particles |  | in small HDL |
|  | small LDL particles |  | in small LDL |
|  | small VLDL particles |  | in small VLDL |
|  | very large HDL particles |  | in very large HDL |
|  | very large VLDL particles |  | in very large VLDL |
|  | very small VLDL particles |  | in very small VLDL |
| **76 Genetic markers** | | | |
| 1:50909985_A | 1:50909985_A | 1:50909985_A | 1:50909985_A |
| 1:120517959_T | 1:120517959_T | 1:120517959_T | 1:120517959_T |
| 1:214154719_T | 1:214154719_T | 1:214154719_T | 1:214154719_T |
| 2:27741237_T | 2:27741237_T | 2:27741237_T | 2:27741237_T |
| 2:43690030_T | 2:43690030_T | 2:43690030_T | 2:43690030_T |
| 2:60568745_A | 2:60568745_A | 2:60568745_A | 2:60568745_A |
| 2:135479980_G | 2:135479980_G | 2:135479980_G | 2:135479980_G |
| 2:151637936_G | 2:151637936_G | 2:151637936_G | 2:151637936_G |
| 2:161171454_T | 2:161171454_T | 2:161171454_T | 2:161171454_T |
| 2:165501849_C | 2:165501849_C | 2:165501849_C | 2:165501849_C |
| 2:227093585_A | 2:227093585_A | 2:227093585_A | 2:227093585_A |
| 3:12393125_G | 3:12393125_G | 3:12393125_G | 3:12393125_G |
| 3:23336450_A | 3:23336450_A | 3:23336450_A | 3:23336450_A |
| 3:64048297_T | 3:64048297_T | 3:64048297_T | 3:64048297_T |
| 3:64705365_C | 3:64705365_C | 3:64705365_C | 3:64705365_C |
| 3:123082398_C | 3:123082398_C | 3:123082398_C | 3:123082398_C |
| 3:185511687_T | 3:185511687_T | 3:185511687_T | 3:185511687_T |
| 3:186666461_T | 3:186666461_T | 3:186666461_T | 3:186666461_T |
| 3:187740523_T | 3:187740523_T | 3:187740523_T | 3:187740523_T |
| **Note:** Categorical variables transformed to dummy ones during analysis. | | | |

| **Supplementary Table 1c. Median AUC [IQR] performance of the ML models using Dataset A - F** | | | | | | |
| --- | --- | --- | --- | --- | --- | --- |
| Model | A: traditional risk factors | B: A + Extended | C: B + Imaging | D: B + Genetic | E: B + Metabolites | F: All 339 Features |
| EN | 0.797  (0.77, 0.828) | 0.827  (0.807, 0.847) | 0.822  (0.803, 0.844) | 0.737  (0.7, 0.767) | **0.843**  **(0.823, 0.862)** | 0.754  (0.721, 0.785) |
| LASSO | 0.781  (0.757, 0.807) | 0.811  (0.788, 0.834) | 0.806  (0.786, 0.825) | 0.773  (0.75, 0.802) | 0.814  (0.791, 0.837) | 0.772  (0.738, 0.801) |
| GBDT | 0.789  (0.762, 0.813) | 0.807  (0.789, 0.828) | 0.807  (0.781, 0.832) | 0.783  (0.752, 0.809) | 0.809  (0.789, 0.832) | 0.779  (0.747, 0.811) |
| LR | 0.796  (0.774, 0.825) | 0.821  (0.8, 0.842) | 0.811  (0.79, 0.831) | 0.58  (0.554, 0.62) | 0.622  (0.59, 0.651) | 0.584  (0.549, 0.628) |
| XGB | 0.764  (0.74, 0.791) | 0.801  (0.78, 0.821) | 0.804  (0.78, 0.826) | 0.77  (0.742, 0.797) | 0.801  (0.781, 0.82) | 0.788  (0.754, 0.811) |
| RF | 0.774  (0.748, 0.795) | 0.814  (0.793, 0.834) | 0.817  (0.793, 0.836) | 0.785  (0.759, 0.806) | 0.772  (0.745, 0.795) | 0.745  (0.71, 0.772) |
| NB | 0.782  (0.756, 0.808) | 0.812  (0.79, 0.836) | 0.793  (0.768, 0.821) | 0.753  (0.722, 0.783) | 0.645  (0.611, 0.679) | 0.657  (0.604, 0.705) |
| CART | 0.702  (0.664, 0.733) | 0.744  (0.711, 0.777) | 0.742  (0.701, 0.772) | 0.666  (0.612, 0.714) | 0.703  (0.652, 0.731) | 0.631  (0.563, 0.701) |
| SVM | 0.604  (0.574, 0.658) | 0.744  (0.722, 0.77) | 0.75  (0.728, 0.783) | 0.722  (0.684, 0.747) | 0.728  (0.7, 0.755) | 0.723  (0.686, 0.751) |

| **Supplementary Table 1d. Median SN%/ SP% performance of the ML models using Dataset A - F** | | | | | | | | | | | | |
| --- | --- | --- | --- | --- | --- | --- | --- | --- | --- | --- | --- | --- |
| Model | A: traditional  risk factors | | B: A + Extended | | C: B + Imaging | | D: B + Genetic | | E: B + Metabolites | | F: All 339 Features | |
| EN | 80.65 | 71.91 | 87.5 | 70 | 87.1 | 69.65 | 75 | 68.85 | **83.87** | **74.79** | 82.61 | 63.37 |
| LASSO | 83.87 | 66.38 | 87.5 | 69.17 | 83.87 | 68.12 | 83.33 | 68.31 | 83.87 | 70.09 | 82.61 | 65.7 |
| GBDT | 80.65 | 71.7 | 81.25 | 70.83 | 83.87 | 69.65 | 83.33 | 67.49 | 85.48 | 69.23 | 82.61 | 68.6 |
| LR | 80.65 | 73.4 | 87.5 | 68.54 | 87.1 | 67.9 | 29.17 | 87.43 | 38.71 | 86.32 | 52.17 | 70.35 |
| XGB | 80.65 | 68.09 | 84.38 | 68.33 | 83.87 | 70.96 | 83.33 | 66.94 | 83.87 | 69.44 | 82.61 | 70.93 |
| RF | 79.03 | 69.15 | 84.38 | 70 | 83.87 | 71.62 | 87.5 | 63.66 | 83.87 | 63.68 | 82.61 | 63.37 |
| NB | 77.42 | 71.49 | 84.38 | 69.17 | 83.87 | 69.21 | 79.17 | 66.12 | 64.52 | 64.96 | 60.87 | 75.87 |
| CART | 70.97 | 66.38 | 75 | 69.58 | 77.42 | 69.21 | 62.5 | 74.86 | 70.97 | 67.95 | 60.87 | 75 |
| SVM | 48.39 | 79.15 | 78.12 | 65.83 | 77.42 | 67.69 | 75 | 66.67 | 77.42 | 63.68 | 80.43 | 63.37 |

| **Supplementary Table 1e. Source data linked to Figure 3*** | | | | | | | |
| --- | --- | --- | --- | --- | --- | --- | --- |
| A: Forest plot for the top variables in Elastic Net | | | B: Forest plot for the top variables in LASSO | | | C: Bar chart for the top variables in GBDT | |
| Variable | OR (95% CI) | P-value | Variable | OR (95% CI) | P-value | Variable | Relative influence |
| Malay (Ref: Indian) | 1.82 (1.01, 3.28) | 0.047 | Malay (Ref: Indian) | 1.29 (0.74, 2.23) | 0.365 | Pulse Pressure | 7.287249 |
| Chinese (Ref: Indian) | 2.57 (1.45, 4.55) | 0.001 | Chinese (Ref: Indian) | 2.07 (1.22, 3.53) | 0.007 | GFR-EPI | 6.4514 |
| Acetate | 0.45 (0.25, 0.82) | 0.008 | House category: Small | 2.12 (1.08, 4.17) | 0.03 | Age | 4.605769 |
| Age | 1.87 (1.46, 2.39) | < 0.001 | GFR-EPI | 0.59 (0.46, 0.74) | < 0.001 | Systolic Blood Pressure | 4.190088 |
| Anti-DM Meds | 2.29 (1.41, 3.72) | 0.001 | Pulse Pressure | 1.2 (0.79, 1.8) | 0.394 | 3-Hydroxybutyrate | 2.739014 |
| Diabetic Retinopathy | 2.3 (1.45, 3.66) | < 0.001 | Age | 1.62 (1.26, 2.08) | < 0.001 | M-HDL-PL % | 2.439406 |
| Systolic Blood Pressure | 1.66 (1.33, 2.07) | < 0.001 | L-LDL-CE % | 0.81 (0.58, 1.12) | 0.203 | Tyrosine | 1.846505 |
| DHA | 0.77 (0.6, 0.99) | 0.042 | Anti-DM Meds | 2.08 (1.28, 3.38) | 0.003 | DHA | 1.795956 |
| GFR-EPI | 0.58 (0.45, 0.74) | < 0.001 | M-HDL-PL % | 1.55 (1.24, 1.93) | < 0.001 | DHAFA | 1.51538 |
| HbA1c | 1.36 (1.09, 1.69) | 0.006 | Diabetic Retinopathy | 2.03 (1.29, 3.19) | 0.002 | Diabetes Duration | 1.340025 |
| Hypertension | 3.11 (1.28, 7.56) | 0.012 | Hypertension | 2.68 (1.01, 7.14) | 0.049 | XS-VLDL-FC % | 1.328608 |
| IDL-CE % | 0.68 (0.54, 0.85) | 0.001 | IDL-C % | 1.02 (0.72, 1.43) | 0.916 | HDL cholesterol | 1.161815 |
| M-HDL-PL % | 1.48 (1.18, 1.86) | 0.001 | HbA1C | 1.33 (1.08, 1.63) | 0.007 | Lactate | 1.46813 |
| M-VLDL-PL% | 1.45 (1.13, 1.84) | 0.003 | Systolic Blood Pressure | 1.41 (0.94, 2.12) | 0.1 | Body Mass Index | 1.073596 |
| S-HDL-FC % | 1.19 (0.94, 1.5) | 0.152 | Anti-HTN Meds | 1.34 (0.82, 2.2) | 0.243 | Diabetic Retinopathy | 1.047174 |
| XL-HDL-CE % | 1.61 (1.23, 2.11) | 0.001 | DHA | 0.8 (0.63, 1.02) | 0.069 |  |  |

*Elastic Net and LASSO variables were passed to multivariable logistic regression models for the OR (95% CI) and p-value estimation. Herein we showed 16 rows for top-15 variables because ethnicity had three levels (Malay, Indian, and Chinese).

| **Supplementary Table 1f. Top ML-selected predictors for incident DKD in each of the 3 ethnic groups by EN and RFE.** | | | | | | | | | |
| --- | --- | --- | --- | --- | --- | --- | --- | --- | --- |
|  | **Malay (n=380)** | | | **Indian (n=648)** | | | **Chinese (n=337)** | | |
|  | **Variable** | **OR (95% CI)** | **P-value** | **Variable** | **OR (95% CI)** | **P-value** | **Variable** | **OR (95% CI)** | **P-value** |
| 1 | GFR-EPI | 0.68 (0.45, 1.01) | 0.054 | GFR-EPI | 0.43 (0.28, 0.64) | <0.001 | GFR-EPI | 0.34 (0.21, 0.55) | <0.001 |
| 2 | Acetate | 0.66 (0.45, 0.95) | 0.027 | Acetate | 0.53 (0.24, 1.17) | 0.115 | Pulse Pressure | 1.56 (1.01, 2.42) | 0.046 |
| 3 | Systolic BP | 1.89 (1.3, 2.73) | 0.001 | Systolic BP | 2.15 (1.46, 3.16) | <0.001 | Anti-DM Meds | 3.4 (1.22, 9.45) | 0.019 |
| 4 | Hypertension | 13.58 (1.45, 126.86) | 0.022 | Anti-HTN Meds | 2.7 (1.04, 6.97) | 0.04 | House category:  Small | 1.64 (1.24, 2.17) | 0.001 |
| 5 | House category:  Large | 0.6 (0.36, 1.01) | 0.053 | Anti-DM Meds | 3.92 (1.32, 11.65) | 0.014 | M-HDL-PL% | 1.57 (1.01, 2.44) | 0.045 |
| 6 | Age | 1.89 (1.27, 2.81) | 0.002 | XS-VLDL-CE% | 1.44 (0.99, 2.08) | 0.056 | Average axial  length (left) | 0.65 (0.38, 1.11) | 0.114 |
| 7 | HbA1c | 1.73 (1.18, 2.52) | 0.005 | XL-HDL-C% | 1.79 (1.2, 2.67) | 0.004 | XL-HDL-FC% | 0.74 (0.53, 1.03) | 0.071 |
| 8 | Diabetic Retinopathy | 4.75 (2.02, 11.18) | <0.001 | 3-Hydroxybutyrate | 1.44 (1.1, 1.89) | 0.009 | Albumin | 0.69 (0.43, 1.11) | 0.125 |
| 9 | IDL-CE % | 0.53 (0.32, 0.88) | 0.015 | Lactate | 0.61 (0.41, 0.9) | 0.014 |  |  |  |
| 10 | S-LDL-C% | 2.08 (1.15, 3.79) | 0.016 | LDL cholesterol | 0.51 (0.31, 0.86) | 0.011 |  |  |  |
| 11 | S-VLDL-PL% | 1.77 (1.09, 2.87) | 0.02 | XXL-VLDL-TG% | 0.58 (0.41, 0.82) | 0.002 |  |  |  |
| 12 | Degree of unsaturation | 0.34 (0.19, 0.61) | <0.001 |  |  |  |  |  |  |
| *Elastic Net variables were passed to multivariable logistic regression models for the OR (95% CI) and p-value estimation. | | | | | | | | | |

| **Supplementary Table 1g. Baseline characteristics of SEED Diabetic participants by ethnicity (n = 1365)** | | | | |
| --- | --- | --- | --- | --- |
| **Characteristics** | **Malays (n = 380)** | **Indians (n = 648)** | **Chinese (n = 337)** | **p-value** |
| Incident CKD, % | 70 (18.4) | 49 (7.6) | 43 (12.8) | <0.001 |
| Age (years), mean SD | 57.28 [51.72, 64.49] | 57.05 [50.56, 64.24] | 61.26 [54.44, 67.72] | <0.001 |
| Sex, female | 212 (55.8) | 296 (45.7) | 159 (47.2) | 0.006 |
| Primary/below education, % | 272 (71.6) | 360 (55.6) | 195 (57.9) | <0.001 |
| Current smoker, % | 65 (17.1) | 75 (11.6) | 49 (14.5) | 0.043 |
| Alcohol consumption, % | 1 (0.3) | 94 (14.6) | 27 (8.0) | <0.001 |
| Hypertension, % | 293 (77.3) | 448 (69.3) | 259 (76.9) | 0.005 |
| Diabetic retinopathy, % | 59 (15.8) | 170 (26.6) | 55 (16.5) | <0.001 |
| Cardiovascular disease, % | 37 (9.7) | 113 (17.4) | 35 (10.4) | <0.001 |
| Duration of diabetes (years) | 1.40 [0.00, 6.05] | 4.33 [0.00, 10.58] | 2.72 [0.00, 9.41] | <0.001 |
| Anti-diabetic medication, % | 192 (50.5) | 415 (64.0) | 196 (58.2) | <0.001 |
| Insulin use, % | 8 (2.1) | 31 (4.8) | 11 (3.6) | 0.092 |
| Body mass index (kg/m^2^) | 27.56 [24.62, 30.57] | 26.79 [24.00, 29.56] | 24.92 [22.99, 27.64] | <0.001 |
| Systolic blood pressure (mm Hg) | 145.50 [132.88, 163.00] | 136.50 [124.50, 148.62] | 138.00 [128.00, 152.00] | <0.001 |
| Diastolic blood pressure (mm Hg) | 79.50 [71.00, 87.00] | 77.00 [70.50, 83.12] | 76.50 [70.00, 83.00] | 0.004 |
| Random blood glucose (mmol/L) | 8.50 [6.10, 12.72] | 8.40 [6.50, 11.50] | 8.40 [6.50, 11.80] | 0.954 |
| HbA1c, % | 7.35 [6.70, 9.15] | 7.00 [6.60, 8.10] | 7.00 [6.60, 7.90] | <0.001 |
| Blood total Cholesterol (mmol/L) | 5.52 [4.82, 6.28] | 4.76 [4.09, 5.58] | 4.86 [4.30, 5.72] | <0.001 |
| Blood HDL Cholesterol (mmol/L) | 1.22 [1.06, 1.43] | 0.98 [0.83, 1.16] | 1.11 [0.93, 1.36] | <0.001 |
| eGFR (mL/min/1.73 m2) | 81.27 [71.43, 95.67] | 92.15 [80.42, 101.47] | 92.93 [80.92, 101.54] | <0.001 |
| Values for categorical variables are presented as number (percentages); values for continuous variables are given as median [IQR]. p-values are given by the χ2-test test or Kruskal-Wallis test.  Abbreviations: HDL, high-density lipoprotein cholesterol; SD, standard deviation; IQL, interquartile range. | | | | |
